# A Multi-Sensor Fusion-Based Localization Method for a Magnetic Adhesion Wall-Climbing Robot

**DOI:** 10.3390/s25165051

**Published:** 2025-08-14

**Authors:** Xiaowei Han, Hao Li, Nanmu Hui, Jiaying Zhang, Gaofeng Yue

**Affiliations:** 1 School of Mechanical Engineering, Shenyang University, Shenyang 110044, China; hanxw@syu.edu.cn; 2 School of Intelligent Science and Information Engineering, Shenyang University, Shenyang 110044, China; huinm@syu.edu.cn (N.H.); zjy1980@syu.edu.cn (J.Z.); 3 School of Cyber Science and Engineering, Xi’an Jiaotong University, Xi’an 710049, China; yuegaofeng1106@163.com

**Keywords:** magnetic adhesion wall-climbing robot, multi-sensor fusion, Extended Kalman Filter (EKF), residual weighting, localization

## Abstract

To address the decline in the localization accuracy of magnetic adhesion wall-climbing robots operating on large steel structures, caused by visual occlusion, sensor drift, and environmental interference, this study proposes a simulation-based multi-sensor fusion localization method that integrates an Inertial Measurement Unit (IMU), Wheel Odometry (Odom), and Ultra-Wideband (UWB). An Extended Kalman Filter (EKF) is employed to integrate IMU and Odom measurements through a complementary filtering model, while a geometric residual-based weighting mechanism is introduced to optimize raw UWB ranging data. This enhances the accuracy and robustness of both the prediction and observation stages. All evaluations were conducted in a simulated environment, including scenarios on flat plates and spherical tank-shaped steel surfaces. The proposed method maintained a maximum localization error within 5 cm in both linear and closed-loop trajectories and achieved over 30% improvement in horizontal accuracy compared to baseline EKF-based approaches. The system exhibited consistent localization performance across varying surface geometries, providing technical support for robotic operations on large steel infrastructures.

## 1. Introduction

With the rapid advancement of automation and intelligent technologies, magnetic adhesion wall-climbing robots have emerged as a novel type of industrial and service robot. Owing to their high mobility on vertical or inclined metal surfaces, they have been widely deployed in tasks such as cleaning, maintenance, and inspection [1,2,3]. These robots are particularly valuable in hazardous environments where direct human intervention is difficult or dangerous, as they can effectively replace manual labor in executing complex and high-risk operations. As a result, achieving precise localization becomes a critical issue to ensure stable and reliable task execution under various challenging conditions [4].

Conventional localization methods often rely on a single type of sensor, such as Inertial Measurement Units (IMUs), Wheel Odometry (Odom), Ultra-WideBand (UWB) systems, or LiDAR. Although these methods may offer satisfactory performance in low-precision scenarios, they show significant limitations in high-accuracy localization tasks [5,6,7]. Furthermore, despite the notable progress in visual-based localization systems, magnetic wall-climbing robots frequently operate in environments with low illumination, dynamic interference, occlusion, and multipath effects. These factors can severely degrade the reliability and robustness of visual sensors, resulting in insufficient localization accuracy for demanding applications [8,9].

In recent years, an increasing number of researchers have focused on multi-sensor fusion algorithms for localization tasks. These algorithms aim to achieve high-precision estimation of spatial states (e.g., position and orientation) by integrating data from multiple positioning sensors, thereby effectively overcoming the limitations of single-sensor systems and significantly improving localization accuracy and robustness [10,11]. For instance, Cao et al. proposed a fusion localization method that combines an Extended Square-root Kalman Filter (ESEKF) and a Vector-based Bayesian Unscented Kalman Filter (VBUKF) for IMU/UWB integration [12], aiming to enhance localization accuracy in underground coal mining environments. By introducing ESEKF and VBUKF, the method improved the system’s adaptability to sensor noise in complex environments and significantly reduced localization errors. Although this approach demonstrated high accuracy and robustness under real-world conditions, its generalizability in highly dynamic or severely occluded environments remains to be further validated and optimized. Cheng et al. developed a multi-sensor fusion-based localization and navigation system for greenhouse mobile robots [13]. By integrating data from various sensors, their system achieved high-accuracy localization and path planning in greenhouse environments, enhancing autonomous mobility in complex agricultural scenarios. However, its performance is still sensitive to sensor calibration accuracy and environmental interference. Yang et al. proposed a centimeter-level indoor 3D localization method for wheeled robots based on multi-sensor fusion [14]. Their approach utilized multiple sensor inputs to estimate both the position and orientation of the robot in three dimensions, effectively meeting the demands of high-precision indoor navigation. It is worth noting, however, that although the method performed well in controlled environments, its adaptability and real-time performance in complex dynamic scenarios still require further investigation and improvement. Zhang et al. proposed an indoor localization method for mobile robots based on factor graph optimization [15], which effectively integrates data from multiple sensors through a factor graph model, enhancing localization accuracy and system robustness in indoor environments. Zhang et al. developed an autonomous localization approach for climbing robots operating on large steel structures [16], which fuses IMU data with information from a fixed RGB-D camera. Their method improved the localization accuracy and autonomy of wall-climbing robots, adapting to the diverse demands of complex steel surfaces. However, its performance is highly sensitive to camera installation position and lighting conditions and may degrade under extreme surface shapes or occlusion. Sun et al. proposed a robot localization system that fuses UWB, IMUs, and wheel odometry data [17]. Targeting the challenges posed by mixed Line-of-Sight/Non-Line-of-Sight (LOS/NLOS) environments, the author designed a multimodal data fusion framework. A multi-sensor observation model was established to incorporate UWB ranging, IMU orientation, and odometry displacement. Through residual detection and classification mechanisms, the system distinguishes between LOS and NLOS signals, dynamically adjusting the weight of UWB measurements in the fusion process. To address the large errors in NLOS UWB signals, an improved observation model and error compensation strategy were introduced for automatic outlier detection and correction. However, the effectiveness of UWB signal discrimination and error compensation largely depends on threshold settings and environmental priors, limiting generalizability and adaptability in highly dynamic or obstructed conditions. Brunacci et al. presented a localization method that combines UWB and magnetic ranging systems [18], achieving higher stability and anti-interference performance, especially in environments where single-sensor methods are insufficient. Nonetheless, the approach is sensitive to changes in the magnetic field and requires complex system integration and calibration. Hao et al. introduced a localization method that integrates camera and UWB data with positional constraints [19]. By leveraging the spatial relationship between the camera and UWB system, the method improved overall accuracy and robustness. However, it requires accurate initial calibration and field-of-view alignment between the two sensors, posing challenges in complex real-world scenarios. Zhang et al. proposed a dynamic window-based UWB-odometry fusion method for indoor localization [20]. By adaptively adjusting the window size, this method enhanced the flexibility and accuracy of data fusion, particularly in rapidly changing indoor environments. Still, issues such as window parameter selection and long-term error accumulation remain concerns in prolonged or high-dynamic operations. Yuan et al. developed a Motion Inertia-estimated Adaptive Kalman Filter (MIAKF) for tunnel localization in mining applications [21]. This method dynamically adjusts filtering parameters based on motion inertia estimation, improving localization accuracy and adaptability in complex underground environments. Ghadimzadeh Alamdari et al. presented a comprehensive review of SLAM methods for infrastructure inspection in GPS-denied environments [22]. Their benchmark showed that LiDAR-based approaches offer high robustness, while the fusion method LVI-SAM achieved the best overall performance by combining visual texture and depth information. Yan et al. proposed a hybrid method for remote sensing scene classification that combines Res2Net and a vision transformer [23]. By designing a cross-layer interaction mechanism, the model effectively captures both local and global semantic features, showing superior performance on multiple open-source datasets. Nguyen et al. developed a bicycle-inspired climbing robot equipped with odometry, IMUs, and visual sensors for efficient steel structure inspection [24]. The robot demonstrated strong adaptability to curved and inclined surfaces, and its multi-sensor fusion strategy ensured reliable localization during magnetic adhesion. Otsuki et al. proposed an autonomous climbing robot integrated with a wireless ultrasonic device (Martlet) for the in situ thickness measurement of steel bridge members [25]. The system combines magnetic adhesion, automated gel-coupling, and onboard TOF signal processing to achieve accurate and repeatable ultrasonic measurements. Experimental results validated its effectiveness in both vertical and inverted positions, demonstrating strong potential for roboticized structural health monitoring.

To address the aforementioned challenges, this study proposes a multi-sensor fusion localization method that integrates data from an IMU, UWB, and Odom. The method is built upon an EKF framework, which combines the high-frequency responsiveness of the IMU, the global ranging capability of UWB, and the incremental displacement information from odometry to form a complementary filtering structure aimed at improving short-term state prediction. Additionally, a geometric residual-weighted UWB ranging model is introduced to refine raw UWB distance measurements. The resulting observations are used to update the EKF, helping to mitigate the impact of multipath effects and sensor noise on localization accuracy. All evaluations in this study are conducted entirely within a simulated environment, enabling a systematic assessment of the proposed method under controlled and reproducible conditions. The performance validations and comparative analyses are based on simulation data that emulate realistic operational scenarios. The overall contributions and innovations of this work are summarized as follows:

(1) To address the common limitations of traditional trilateration methods under non-line-of-sight (NLOS) and multipath conditions, a geometric residual-weighted UWB ranging model is established. Compared to the conventional trilateration principle, this model integrates multiple sets of residuals to improve the robustness and reliability of raw UWB ranging measurements.
(2) To mitigate zero-bias drift and slip errors in IMU and wheel odometry, a dynamically weighted complementary filtering scheme is designed based on traditional complementary filter principles. This contributes to the short-term pose prediction stability, particularly in dynamic and low-speed motion scenarios.
(3) Development and implementation of a multi-source fusion EKF framework: In the prediction stage, the short-term state estimate is generated by dynamically fusing IMU and wheel odometry through the complementary filter. In the update stage, the optimized UWB ranging data, processed via residual weighting, is introduced as the observation input to the EKF. This supports global state estimation and improves error correction.

## 2. Overview of Localization Algorithms and Sensor Principles

### 2.1. IMU-Based Localization Principles

The IMU comprises two primary sensors: an accelerometer and a gyroscope. Its localization principle is based on dead reckoning algorithms [26], wherein the robot’s attitude is estimated by integrating the angular velocity measured by the gyroscope, while linear velocity is obtained by integrating the acceleration measured by the accelerometer (which includes the effect of gravity), and position is further derived through a second integration.

Although the IMU provides accurate short-term motion measurements, it is susceptible to cumulative errors over time due to sensor bias b and noise n [27]. These errors lead to growing drift in position and orientation estimates during long-term operations. The state variables of the IMU-based localization system are defined as follows:

(1) $$x=\left[\begin{array}{c} p\\ v\\ q\\ b\\ n \end{array}\right]$$

where p is the position in the navigation frame, v is the velocity in the navigation frame, q is the attitude quaternion representing the rotation from the IMU frame to the navigation frame, b is the sensor bias, and n is the white noise of the sensor measurements.

IMU Error Measurement Model:

(2) $$\omega_{m}=\omega_{\mathrm{true}}+b_{\omega }+n_{\omega }$$

(3) $$a_{m}=R^{T}\left(a_{\mathrm{true}}-g\right)+b_{a}+n_{a}$$

where $\omega_{m}$ is the measured angular velocity, $\omega_{true}$ is the true angular velocity, $b_{\omega }$ denotes the gyroscope bias, and $n_{\omega }$ represents the gyroscope noise. $a_{m}$ is the measured acceleration in the IMU coordinate frame, $a_{true}$ is the true acceleration in the navigation coordinate frame, and $b_{a}$ and $n_{a}$ denote the accelerometer bias and noise, respectively. $R^{T}$ is the rotation matrix that transforms vectors from the navigation frame to the IMU frame, and *g* is the constant gravity vector in the navigation frame.

Assuming that at time step k, the IMU provides the robot’s position $p_{k}$, velocity $v_{k}$, angular velocity $\omega_{k}$, accelerometer bias $b_{a}^{k}$, and gyroscope bias $b_{\omega }^{k}$, the discrete-time state transition equations can be established after a time interval ∆t.

Position Update Equation:

(4) $$p_{k+1}=p_{k}+v_{k}\Delta t+\frac{1}{2}\left(R_{k}(a_{m}^{k}-b_{a}^{k}-n_{a}^{k})+g\right)\Delta t^{2}$$

Velocity Update Equation:

(5) $$v_{k+1}=v_{k}+\left(\begin{array}{c} R_{k}(a_{m}^{k}-b_{a}^{k}-n_{a}^{k})+g \end{array}\right)\Delta t$$

Definition of Angular Velocity Increment:

(6) $$\Delta \theta =(\omega_{m}^{k}-b_{\omega }^{k}-n_{\omega }^{k})\Delta t$$

Based on the aforementioned state update and angular increment modeling, the IMU can continuously estimate the motion state of the carrier. The key advantage of the IMU lies in its high-frequency and continuous dynamic response, which enables it to independently estimate both position and orientation over short durations. This makes it particularly suitable for scenarios involving rapid movements. However, the IMU also suffers from significant limitations: due to inherent random drifts and systematic errors in acceleration and angular velocity measurements, cumulative integration leads to rapid error accumulation over time. This manifests as bias drift and noise accumulation, making standalone IMU-based localization unreliable for long-term high-precision applications. Therefore, this paper incorporates external localization sources such as UWB and wheel odometry, aiming to improve the overall positioning accuracy and robustness through multi-sensor data fusion.

### 2.2. Overview of Wheel Odometry-Based Position Estimation

The localization principle of wheel odometry is based on motor encoders mounted on the wheels, which measure the linear and angular velocity of the robot at a certain moment. According to the pose state of the previous moment, the pose of the robot after a time step ∆t can be estimated. During this time step, the robot’s pose trajectory can be approximated as an arc trajectory [28].

For a four-wheeled magnetic adhesion wall-climbing robot, it is assumed that each wheel has a corresponding rotation distance ∆t (i = 1 to 4), where the rotation distance represents the moving distance of each wheel during the time step ∆t [29]. If the pose information of the robot is sampled at time step k, the pose state and the wheel travel distances are as follows:

(7) $$\left[\begin{array}{c} x(k)\\ y(k)\\ \theta (k) \end{array}\right]$$

(8) $$\Delta r_{i}\left(k\right)=N_{i}(k)\times q_{ni}$$

where x(k), y(k), and θ(k) represent the X-coordinate, Y-coordinate, and heading angle of the robot at time step k, respectively. $N_{i}$ denotes the number of encoder pulses recorded by the ith wheel, and $q_{ni}$ is the encoder scale factor of the ith wheel.

The global coordinates of the robot are updated based on the incremental displacement of the wheels as follows:

(9) $$\left\{\begin{array}{c} x(k+1)=x(k)+\Delta x(k)\\ y(k+1)=y(k)+\Delta y(k)\\ \theta (k+1)=\theta (k)+\Delta \theta (k) \end{array}\right.$$

However, in practical applications, the pose estimation of wheel odometry is inevitably affected by error accumulation. The main sources of error include deviations in wheel displacement caused by slippage or uneven ground surfaces, encoder measurement errors, and electronic noise. Moreover, long-term integration further aggravates the accumulation of drift error. Although wheel odometry can provide relatively stable velocity and pose estimation in the short term, its accuracy gradually degrades over time, making it difficult to meet the demands of long-term high-precision localization.

To address this issue, this study integrates wheel odometry and inertial measurements from the IMU through complementary filtering [30,31] and further incorporates UWB observations into an Extended Kalman Filter (EKF) framework for comprehensive state estimation.

### 2.3. Overview of UWB Positioning Techniques

In a UWB positioning system, the 3D coordinates of each base station are known in advance. The system measures the distance between the tag and the base stations by calculating the round-trip time delay of the radio signal transmitted between them [32]. Due to the extremely high time resolution of UWB, which enables sub-nanosecond ranging accuracy, it is particularly suitable for high-precision positioning tasks in complex structural environments [33].

**Figure 1:**
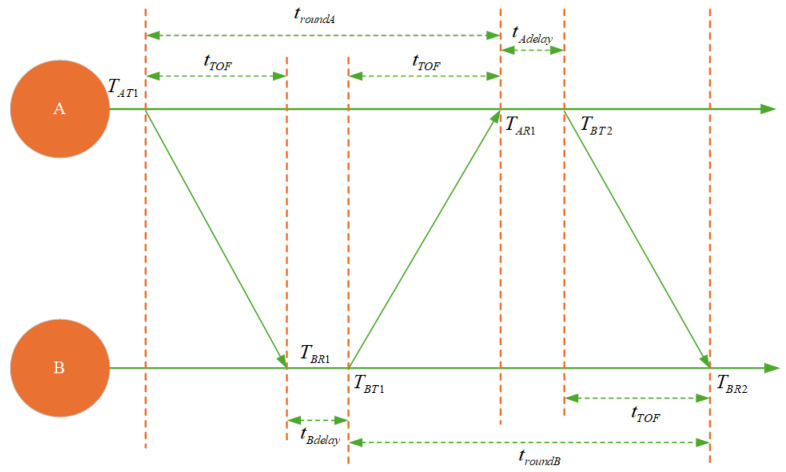
Double-sided two-way ranging.

UWB systems commonly adopt the Double-Sided Two-Way Ranging (DS-TWR) protocol, as shown in Figure 1. This protocol estimates the round-trip propagation time between the tag and base stations through multiple time-delay exchanges between nodes, thereby reducing the impact of synchronization errors and improving ranging accuracy [34].

Let the Time of Flight (TOF) between the tag and the anchor be denoted as $t_{TOF}$. Then, the corresponding distance can be calculated as:

(10) $$d_{i}=c\cdot t_{TOF}^{i}$$

where $d_{i}$ is the measured distance from the ith anchor to the tag, and c is the speed of light.

**Figure 2:**
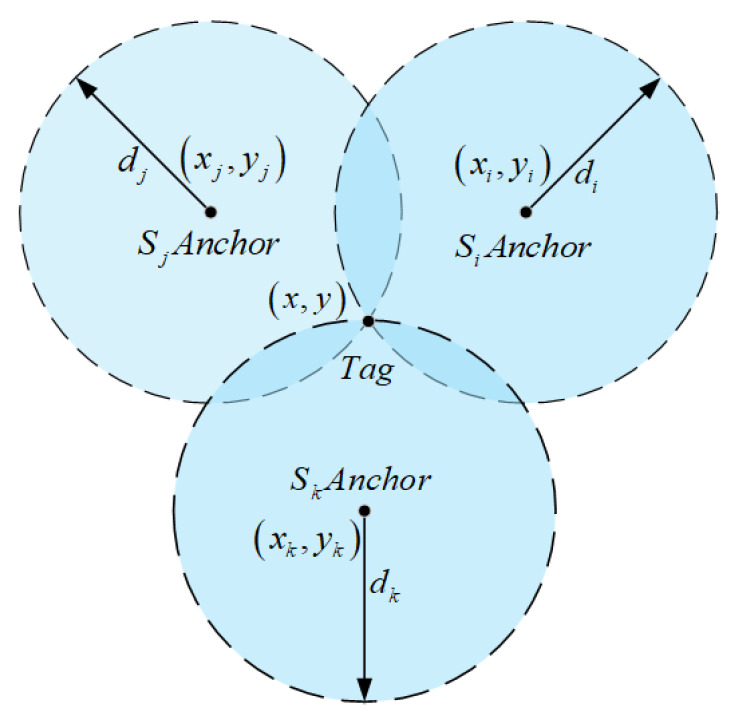
Traditional trilateration method.

To achieve high-precision estimation of the unknown tag position $\left(x,y\right)$, the system typically deploys multiple non-collinear anchors $S_{i}\left(x_{i},y_{i}\right)$ and measures the distances $d_{i}$ between each anchor and the tag. Taking a conventional three-anchor system as an example, three anchors $S_{i}\left(x_{i},y_{i}\right)$, $S_{j}\left(x_{j},y_{j}\right)$, and $S_{k}\left(x_{k},y_{k}\right)$ are selected, as shown in Figure 2.

Based on this geometric relationship, a trilateration equation system can be established:

(11) $${\left(x-x_{m}\right)}^{2}+{\left(y-y_{m}\right)}^{2}=d_{m}^{2},\;\;\;m\in \{i,j,k\}$$

To eliminate the squared terms and transform the problem into a linear solution form, pairwise differencing between any two base stations yields the following system of equations:

(12) $$\left\{\begin{array}{c} 2(x_{i}-x_{k})x+2(y_{i}-y_{k})y=x_{i}^{2}-x_{k}^{2}+y_{i}^{2}-y_{k}^{2}+d_{k}^{2}-d_{i}^{2}\\ 2(x_{j}-x_{k})x+2(y_{j}-y_{k})y=x_{j}^{2}-x_{k}^{2}+y_{j}^{2}-y_{k}^{2}+d_{k}^{2}-d_{j}^{2} \end{array}\right.$$

Further arranging the equations into matrix form yields:

(13) AX=b

(14) $$A=\left[\begin{array}{cc} 2(x_{i}-x_{k}) & 2(y_{i}-y_{k})\\ 2(x_{j}-x_{k}) & 2(y_{j}-y_{k}) \end{array}\right]$$

(15) $$b=\left[\begin{array}{c} x_{i}^{2}-x_{k}^{2}+y_{i}^{2}-y_{k}^{2}+d_{k}^{2}-d_{i}^{2}\\ x_{j}^{2}-x_{k}^{2}+y_{j}^{2}-y_{k}^{2}+d_{k}^{2}-d_{j}^{2} \end{array}\right]$$

Finally, after processing, a linear system of equations is obtained, from which the coordinate information of the tag $\left(x,y\right)$ can be calculated.

(16) $$\left(\begin{array}{c} x\\ y \end{array}\right)=X=A^{-1}b$$

The above represents the formula derivation of the traditional trilateration algorithm. However, this method has good scalability and can be extended to a multi-directional positioning system with any number of anchors, as long as the number of anchors N≥3 and their distribution is non-collinear. Its general form can be expressed as:

(17) $$A=\left[\begin{array}{cc} 2(x_{1}-x_{N}) & 2(y_{1}-y_{N})\\ 2(x_{2}-x_{N}) & 2(y_{2}-y_{N})\\ \vdots & \vdots \\ 2(x_{N-1}-x_{N}) & 2(y_{N-1}-y_{N}) \end{array}\right]$$

(18) $$b=\left[\begin{array}{c} x_{1}^{2}-x_{N}^{2}+y_{1}^{2}-y_{N}^{2}+d_{N}^{2}-d_{1}^{2}\\ x_{2}^{2}-x_{N}^{2}+y_{2}^{2}-y_{N}^{2}+d_{N}^{2}-d_{2}^{2}\\ \vdots \\ x_{N-1}^{2}-x_{N}^{2}+y_{N-1}^{2}-y_{N}^{2}+d_{N}^{2}-d_{N-1}^{2} \end{array}\right]$$

Although the trilateration method offers advantages such as computational simplicity and strong real-time performance, its positioning accuracy is highly sensitive to the geometric distribution of anchors and ranging errors [35]. When the ranging values are affected by multipath interference, occlusions, or noise, the method is prone to significant errors, which can impact the overall stability of the system [36].

To enhance the robustness and effectiveness of UWB ranging observations in the multi-sensor fusion algorithm, this paper proposes a residual-weighted optimization based on the traditional trilateration method. By dynamically assigning weights according to the residuals between the predicted position and the measured distances from each anchor, the system performs a weighted correction of the trilateration process, resulting in an optimized UWB position estimate. The proposed method retains the geometric foundation of traditional trilateration while improving its resilience to abnormal ranging values and enhancing global positioning stability, thereby providing a more reliable multi-source fusion input for the robotic system.

## 3. Fusion Localization Algorithm Based on IMU-Odom-UWB

To improve localization precision and maintain reliable system performance of the magnetically adhered wall-climbing robot in large-scale steel structure environments, this paper proposes a multi-sensor fusion localization algorithm based on the EKF. The proposed method integrates the high-frequency dynamic response of the IMU, the short-term displacement increments from the wheel odometry, and the global ranging information from the UWB system to estimate the robot’s pose. We also considered the potential impact of magnetic field interference on the IMU, particularly the magnetometer, and designed the system to reduce reliance on the IMU alone, thereby mitigating the drift caused by magnetic disturbances.

**Figure 3:**
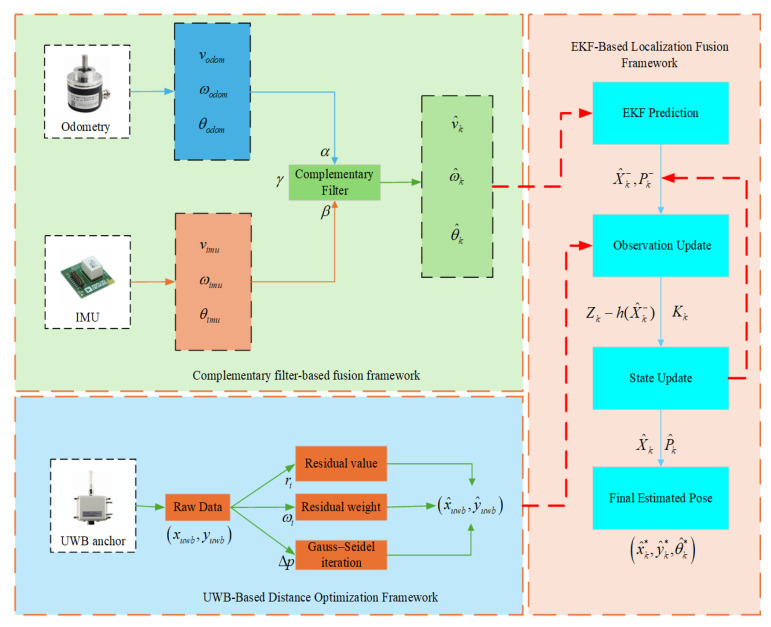
Framework of the proposed localization algorithm.

The overall algorithm consists of key modules such as complementary filtering fusion, UWB ranging optimization, and EKF state update. The Extended Kalman Filter (EKF) framework allows for dynamic adjustment of the sensor fusion process, improving localization robustness. The dynamic complementary filtering method, with adjustable weighting factors, further reduces the trust in the IMU data when they are compromised by magnetic interference. Optimized UWB data help correct localization errors, enhancing accuracy. The structured processing flow is illustrated in Figure 3, and the following subsections provide detailed derivations and explanations of the main algorithmic components.

### 3.1. Establishment of a Geometric Residual Weighted UWB Ranging Model

In a typical UWB positioning system, the position $\left(x,y\right)$ of the target node is usually determined by solving a traditional trilateration model using the measured distances $d_{i}$ between the target and N anchor nodes located at $\left(x_{i},y_{i}\right)$, which satisfies:

(19) $$J_{i}=\left[\frac{x-x_{i}}{\sqrt{{\left(x-x_{i}\right)}^{2}+{\left(y-y_{i}\right)}^{2}}}\right.,\left.\frac{y-y_{i}}{\sqrt{{\left(x-x_{i}\right)}^{2}+{\left(y-y_{i}\right)}^{2}}}\right]$$

**Figure 4:**
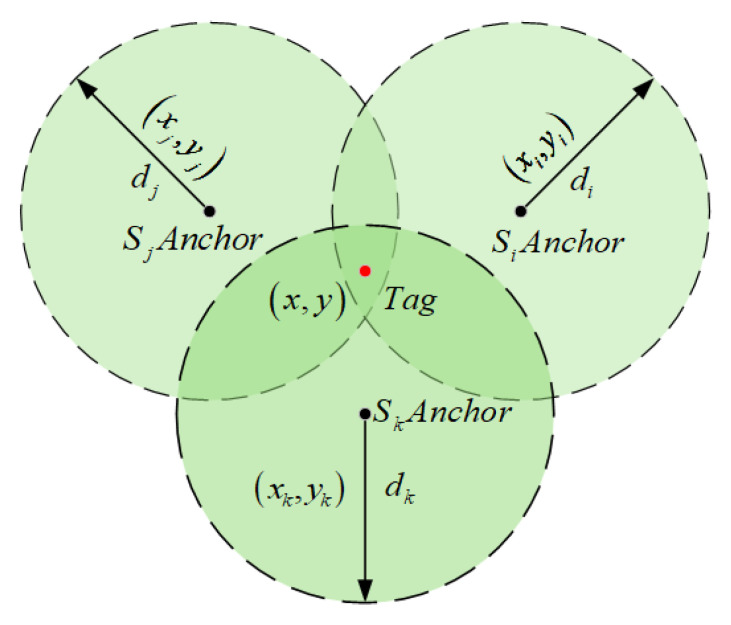
Illustration of error deviation in traditional trilateration.

In theory, the intersection point of the three circles represents the position of the tag. However, in real-world complex environments, signal propagation often deviates from the ideal free-space model. Factors such as non-line-of-sight (NLOS) conditions, wall obstructions, metal reflections, and multipath interference can introduce noticeable deviations in the measured distances $d_{i}$. As a result, the ranging circles may fail to intersect at a single point, leading to positioning inaccuracies, as illustrated in Figure 4.

To address this issue, a geometric residual weighting mechanism is introduced in this paper to optimize UWB ranging. The ranging residual is constructed as follows:

(20) $$r_{i}=\sqrt{{\left(x-x_{i}\right)}^{2}+{\left(y-y_{i}\right)}^{2}}-d_{i}$$

Then, a weighted least squares objective function is constructed as follows:

(21) $$J(x,y)=\sum_{i=1}^{N}w_{i}r_{i}^{2}=\sum_{i=1}^{N}w_{i}{\left(\sqrt{{\left(x-x_{i}\right)}^{2}+{\left(y-y_{i}\right)}^{2}}-d_{i}\right)}^{2}$$

Here, the weight $w_{i}$ is used to suppress the influence of large residual observations, thereby reducing the risk of interference caused by NLOS or multipath signals. To improve the performance and stability of the filtering update, a dynamic weighting mechanism based on residual variation is further introduced. In addition, a time-related weighting factor is incorporated to adjust the observation noise covariance matrix.

(22) $$w_{i}=\frac{1}{r_{i}^{2}+\varepsilon },\;\;\;\varepsilon >0$$

where $\varepsilon =10^{-6}$ is a small positive constant introduced to prevent division by zero.

To avoid the nonlinear solution from falling into a local optimum, the Gauss–Newton method is adopted for iterative optimization, and the position estimate is updated as:

(23) $$X^{\left(k+1\right)}=X^{\left(k\right)}-{\left(J^{T}WJ\right)}^{-1}J^{T}Wr$$

where $X^{k}={\left[x^{\left(k\right)},y^{\left(k\right)}\right]}^{T}$ is the position estimate at the kth iteration; $W=diag\left(w_{1},w_{2},\ldots ,w_{N}\right)$ is the diagonal weight matrix; r is the residual vector; and J is the Jacobian matrix, where the ith row of J is the row vector composed of the partial derivatives of the residual $r_{i}$ with respect to x and y, that is:

(24) $$J_{i}=\left[\frac{\partial r_{i}}{\partial x},\frac{\partial r_{i}}{\partial y}\right]$$

The computation is as follows:

(25) $$\frac{\partial r_{i}}{\partial x}=\frac{x-x_{i}}{\sqrt{{\left(x-x_{i}\right)}^{2}+{\left(y-y_{i}\right)}^{2}}},\frac{\partial r_{i}}{\partial y}=\frac{y-y_{i}}{\sqrt{{\left(x-x_{i}\right)}^{2}+{\left(y-y_{i}\right)}^{2}}}$$

(26) $$J_{i}=\left[\frac{x-x_{i}}{\sqrt{{\left(x-x_{i}\right)}^{2}+{\left(y-y_{i}\right)}^{2}}},\frac{y-y_{i}}{\sqrt{{\left(x-x_{i}\right)}^{2}+{\left(y-y_{i}\right)}^{2}}}\right]$$

The final position estimate obtained after iterative convergence is used as the observation input for the EKF. Through the aforementioned geometric residual weighting optimization, the robustness of UWB ranging under dynamic occlusion and multipath interference is significantly enhanced, thereby providing a more reliable observation foundation for subsequent multi-sensor fusion.

### 3.2. Design of a Dynamically Weighted Complementary Filter

To improve the reliability of position estimation during short-term climbing operations of the magnetically adhered wall-climbing robot, this study introduces a complementary filtering method that fuses data from the IMU and wheel odometry. This approach facilitates time–domain cooperative estimation across multiple sensor sources by utilizing the high-frequency responsiveness of the IMU and the accumulated motion information of the wheel odometry under low-speed climbing conditions. A dynamically weighted complementary filter framework is adopted to enable adaptive fusion of these inputs.

Let the state variables of the robot in the navigation coordinate frame be defined as follows:

(27) $$X_{k}={\left[\begin{array}{ccc} x_{k} & y_{k} & \theta_{k} \end{array}\right]}^{T}$$

where $x_{k}$, $y_{k}$ denote the position on a 2D plane, and $\theta_{k}$ represents the heading angle. The system state satisfies the following nonlinear motion equations in continuous time:

(28) $$\dot{X}(t)=\left[\begin{array}{c} \dot{x}(t)\\ \dot{y}(t)\\ \dot{\theta }(t) \end{array}\right]=\left[\begin{array}{c} v(t)\cos\theta (t)\\ v(t)\sin\theta (t)\\ \omega (t) \end{array}\right]$$

(29) $$f_{k}=\left[\begin{array}{c} v_{k}\cos\theta_{k}\\ v_{k}\sin\theta_{k}\\ \omega_{k} \end{array}\right]$$

where $v_{k}$ is the estimated linear velocity of the robot, $\omega_{k}$ is the angular velocity, t is the sampling period, and $W_{k}$ is the process noise, which follows a zero-mean Gaussian distribution.

Construction of the Complementary Filter:

Due to the integration drift issue of the IMU and the susceptibility of wheel odometry to wheel slip interference, this paper performs complementary fusion of the estimations from both sensors. The fused velocity is defined as:

(30) $${\hat{v}}_{k}=\alpha_{k}v_{k}^{imu}+(1-\alpha_{k})v_{k}^{odom}$$

(31) $${\hat{\omega }}_{k}=\beta_{k}\omega_{k}^{imu}+(1-\beta_{k})\omega_{k}^{odom}$$

(32) $${\hat{\theta }}_{k}=\gamma_{k}\theta_{k}^{imu}+(1-\gamma_{k})\theta_{k}^{odom}$$

where $v_{k}^{imu}$, $\omega_{k}^{imu}$, and $\theta_{k}^{imu}$ represent the linear velocity, angular velocity, and orientation angle estimated by the IMU, and $v_{k}^{odom}$, $\omega_{k}^{odom}$, and $\theta_{k}^{odom}$ are the corresponding values computed by the wheel odometry. The parameters $\alpha_{k}$, $\beta_{k}$, and $\gamma_{k}$ are the weighting factors in the complementary filter for the velocity and angular velocity channels, representing the degree of trust in the IMU and wheel odometry data, respectively.

**Figure 5:**
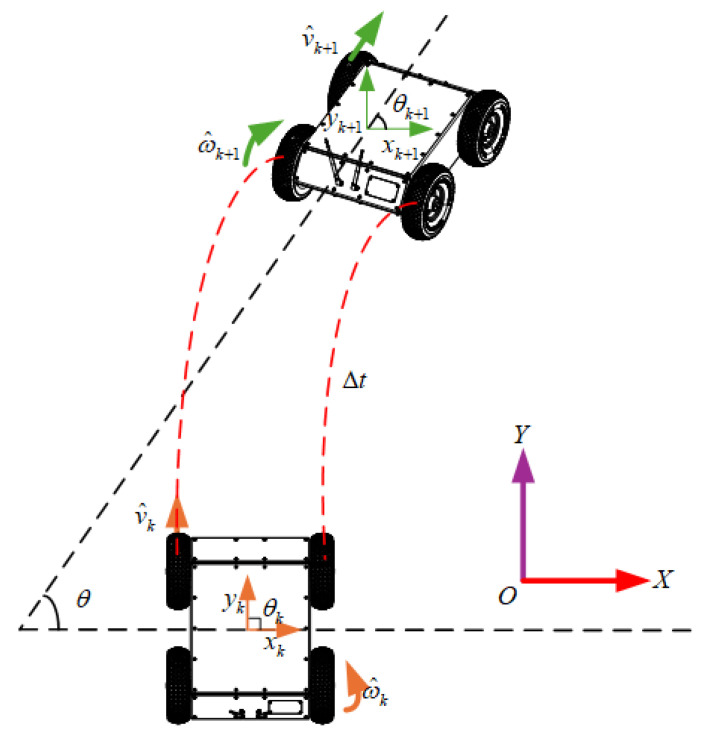
Illustration of pose prediction based on IMU and odometer data.

To provide a more intuitive understanding of the data fusion process between the two sensors and the prediction step, the following figure illustrates the pose prediction of a wheeled robot based on IMU and odometer data, as shown in Figure 5:

State Update and Pose Prediction:

By substituting the fused velocity estimates into the motion prediction model, the robot’s pose at the next time step can be updated as:

(33) $$x_{k+1}=x_{k}+{\hat{v}}_{k}\cos\theta_{k}\cdot \Delta t$$

(34) $$y_{k+1}=y_{k}+{\hat{v}}_{k}\sin\theta_{k}\cdot \Delta t$$

(35) $$\theta_{k+1}=\theta_{k}+{\hat{\omega }}_{k}\cdot \Delta t$$

The above equations provide the state prediction results after complementary fusion of IMU and odometer data, serving as the time prediction input for the subsequent EKF.

### 3.3. EKF Fusion Modeling and State Estimation Method

To achieve accurate fusion of multi-source heterogeneous data in the spatiotemporal domain, this paper constructs a nonlinear state estimation model based on the EKF, using the prediction results from the complementary filter and the optimized UWB ranging data. In this method, the estimates from the IMU and wheel odometry are used as the system’s state prediction input, while the optimized UWB localization results serve as the observation input, thereby enabling dynamic correction and real-time estimation of the state of the magnetically adhered wall-climbing robot. Building upon the short-term motion prediction provided by IMU and odometry fusion, the periodically introduced UWB measurements serve to mitigate the effects caused by incremental drift. By regularly anchoring the estimated states to an absolute spatial reference, the EKF update process contributes to reducing long-term error accumulation and maintaining consistency throughout extended operation.

State Definition and System Modeling

Based on the defined motion state of the robot, the system state vector is expressed as:

(36) $$X_{k}={\left[\begin{array}{ccccc} x_{k} & y_{k} & \theta_{k} & v_{k} & \omega_{k} \end{array}\right]}^{T}$$

where $\left(x_{k},y_{k}\right)$ denotes the position of the robot in the world coordinate system, $\theta_{k}$ is the heading angle, $v_{k}$ is the linear velocity, and $\omega_{k}$ is the angular velocity.

Considering the motion state prediction of the system at discrete time step k, and incorporating the fused estimates ${\hat{v}}_{k}$ and ${\hat{\omega }}_{k}$ from the complementary filter, the state transition function f is formulated as follows:

(37) $$x_{k|k-1}=f(x_{k-1},u_{k})=\left[\begin{array}{c} x_{k-1}+{\hat{v}}_{k}\cos\theta_{k-1}\Delta t\\ y_{k-1}+{\hat{v}}_{k}\sin\theta_{k-1}\Delta t\\ \theta_{k-1}+{\hat{\omega }}_{k}\Delta t\\ {\hat{v}}_{k}\\ {\hat{\omega }}_{k} \end{array}\right]$$

where $u_{k}=\left[{\hat{v}}_{k},{\hat{\omega }}_{k}\right]$ represents the control input derived from the complementary filter output, and ∆t is the discrete sampling interval.

The state transition Jacobian matrix $F_{k}=\partial f/\partial x$ is given by the following:

(38) $$F_{k}=\left[\begin{array}{ccccc} 1 & 0 & -{\hat{v}}_{k}\sin\theta_{k-1}\Delta t & \cos\theta_{k-1}\Delta t & 0\\ 0 & 1 & {\hat{v}}_{k}\cos\theta_{k-1}\Delta t & \sin\theta_{k-1}\Delta t & 0\\ 0 & 0 & 1 & 0 & \Delta t\\ 0 & 0 & 0 & 0 & 0\\ 0 & 0 & 0 & 0 & 0 \end{array}\right]$$

The system process noise covariance matrix $Q_{k}$ is composed based on the uncertainties in the linear and angular velocities provided by the IMU and odometry.

Construction of the Observation Model

The observation obtained from the UWB positioning after geometric residual optimization is denoted as:

(39) $$Z_{k}={\left[\begin{array}{ccc} {\hat{x}}_{uwb} & & {\hat{y}}_{uwb} \end{array}\right]}^{T}$$

Construct the observation model function as:

(40) $$Z_{k}=h(X_{k})=\left[\begin{array}{c} x_{k}\\ y_{k} \end{array}\right]$$

The observation Jacobian matrix is as follows:

(41) $$H_{k}=\frac{\partial h}{\partial X}=\left[\begin{array}{ccccc} 1 & 0 & 0 & 0 & 0\\ 0 & 1 & 0 & 0 & 0 \end{array}\right]$$

The observation noise covariance matrix $R_{k}$ is constructed based on the dynamically weighted residuals of UWB ranging measurements, reflecting the fluctuation level of UWB errors at each time step.

Filtering Prediction and Update Process

Prediction Step:

(42) $${\hat{X}}_{k}^{-}=f({\hat{X}}_{k-1},u_{k})$$

(43) $${\hat{P}}_{k}^{-}=F_{k}{\hat{P}}_{k-1}F_{k}^{T}+Q_{k}$$

Update Step:

(44) $$K_{k}=P_{k}^{-}H_{k}^{T}{\left(H_{k}P_{k}^{-}H_{k}^{T}+R_{k}\right)}^{-1}$$

(45) $${\hat{X}}_{k}={\hat{X}}_{k}^{-}+K_{k}(Z_{k}-h({\hat{X}}_{k}^{-}))$$

(46) $${\hat{P}}_{k}=(I-K_{k}H_{k}){\hat{P}}_{k}^{-}$$

where $K_{k}$ is the Kalman gain, and ${\hat{X}}_{k}$ is the optimally fused state estimate.

**Figure 6:**
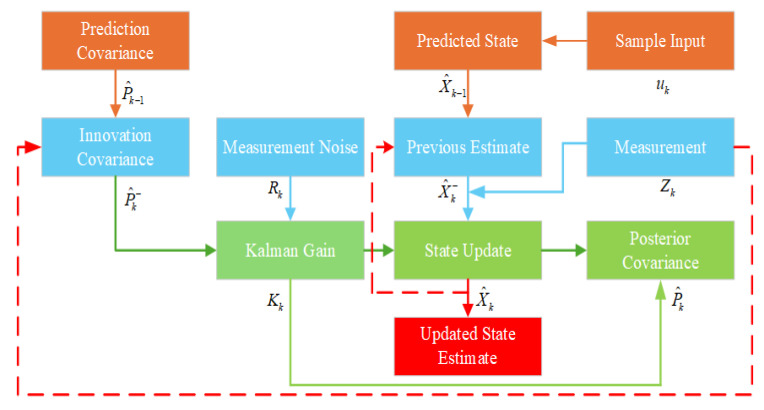
Prediction and update process of the EKF-based fusion algorithm.

To summarize, the following diagram in Figure 6 outlines the prediction and update process of the EKF-based fusion framework.

## 4. Results and Discussion

To evaluate the performance and robustness of the proposed multi-sensor fusion localization method based on an IMU, odometry, and UWB in large-scale steel wall environments, a simulation environment was established on the MATLAB platform. All simulation data were generated based on a predefined trajectory: the synthesized motion states of the robot at each time step (including position, velocity, and orientation) were first created and then combined with physical and noise models of various sensors to simulate observations and generate noisy sensor data.

During the simulation, the sampling frequencies of the IMU, wheel odometry, and UWB ranging were set to 100 Hz, 20 Hz, and 10 Hz, respectively. The main simulation step size was set to 0.01 s. All types of data were generated at their respective frequencies and synchronized before being input into the fusion algorithm to ensure temporal consistency.

To facilitate simulation and ensure clarity of presentation, the simulation setup adopts a standard four-corner anchor layout. However, it should be noted that the proposed residual-weighted UWB optimization algorithm is not inherently dependent on any specific anchor geometry. The approach remains applicable under flexible and irregular anchor distributions, which are often encountered in industrial environments, such as curved tanks, steel trusses, or obstructed wall surfaces. This flexibility enables adaptable deployment without algorithmic modification.

**Figure 7:**
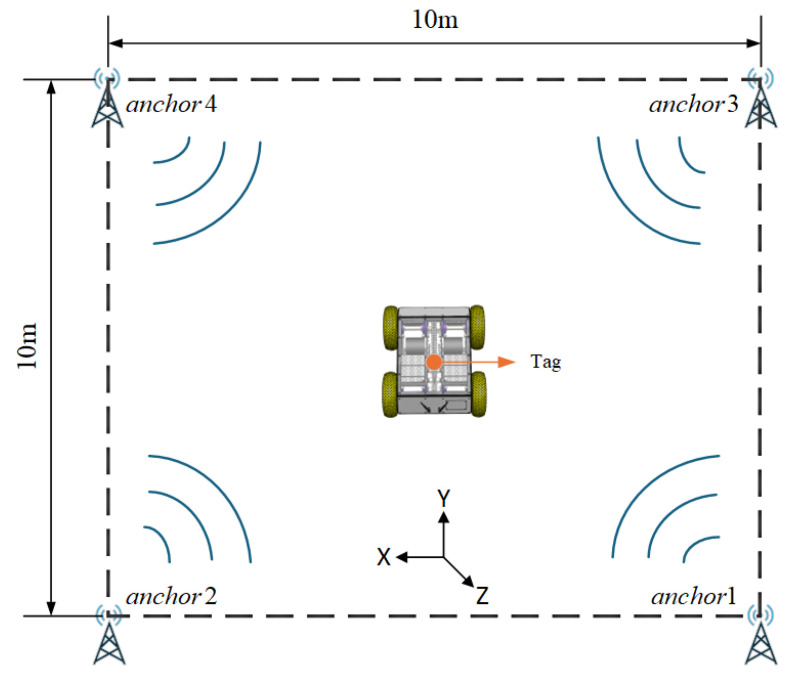
Virtual simulation environment layout.

To better illustrate the layout of the simulation environment, a virtual scene was drawn and is shown in Figure 7. It depicts a 10 m × 10 m vertical wall surface, with four UWB anchors ideally placed at the corners. The robot follows a predefined trajectory while collecting various types of sensor data in real time.

Two sets of simulation tests were conducted during the simulation phase: a straight-line trajectory and a closed rectangular trajectory. In the straight-line simulation, the robot’s initial position was set to (5, 2) meters, with the initial heading along the positive Y-axis. In the rectangular trajectory experiment, the starting point was (1, 1) meters, with the initial heading along the positive X-axis. All experiments were configured with an initial linear velocity of 0.1 m/s and an initial angular velocity of 0 rad/s. The robot followed the predefined trajectory, and various sensor observation data were generated by combining the ideal trajectory with noise models. All sensor noise was modeled as zero-mean Gaussian white noise. The main parameters used in the simulation are listed in Table 1.

### 4.1. Straight-Line Trajectory Localization Simulation

To evaluate the positioning accuracy of the magnetic adhesion wall-climbing robot during a vertical single-axis climbing task, the robot was set to move vertically upward from position (5, 2) to (5, 8) along the Y-axis at a constant speed. This forms a typical vertical motion path used to assess the system’s ability to suppress lateral drift and track longitudinal displacement under conditions without turning or lateral disturbances.

**Figure 8:**
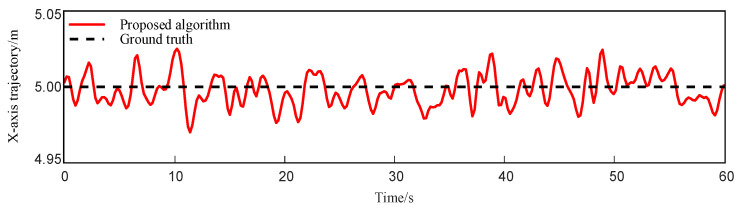
X-Axis trajectory tracking performance.

As shown in Figure 8, the trajectory deviation in the lateral (X-axis) direction remains within ±5 cm throughout the robot’s movement along the X = 5 m line.

**Figure 9:**
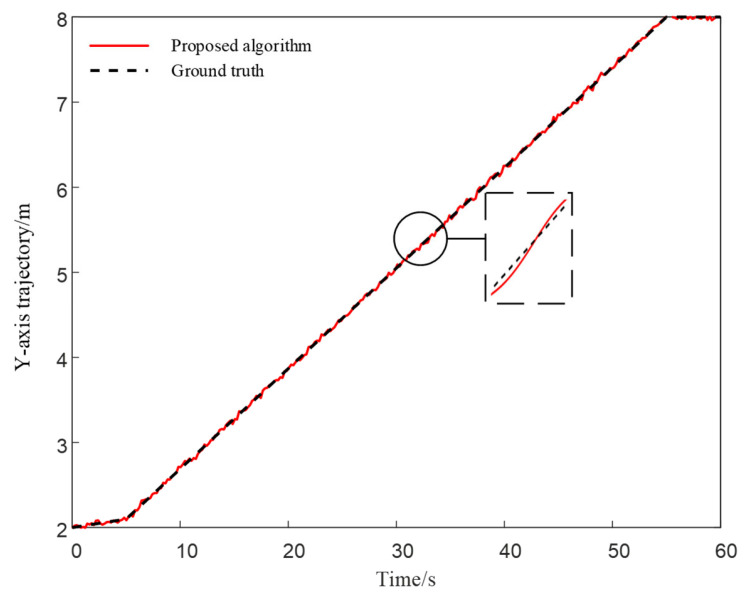
Y-axis trajectory tracking performance. The magnified inset applies approximately 15× vertical scaling relative to the main plot to improve the visibility of local trajectory fluctuations.

**Figure 10:**
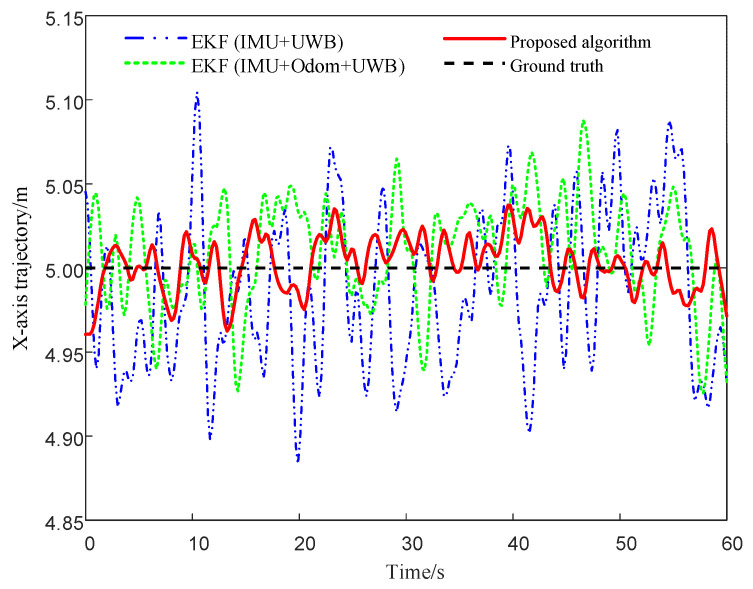
Comparison of X-axis trajectories under different algorithms.

**Figure 11:**
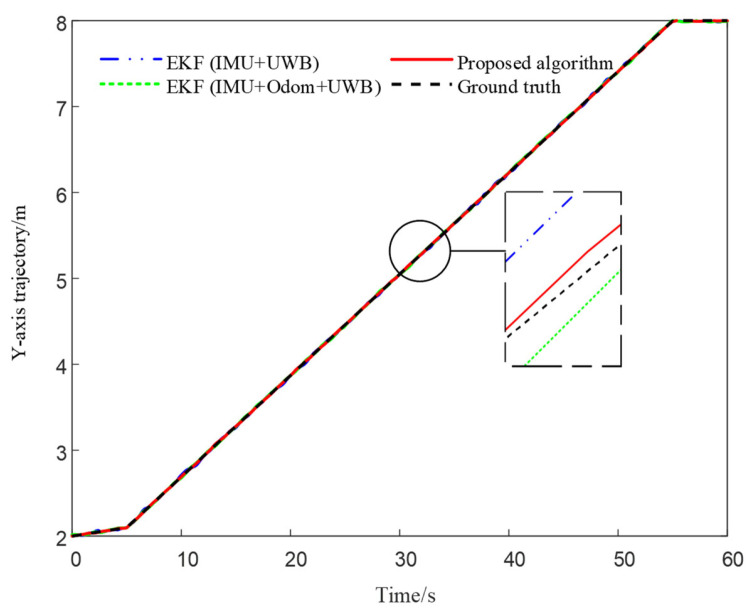
Comparison of Y-axis Trajectories Under Different Algorithms. The inset similarly uses vertical magnification (~15×) for better visibility of fine-scale deviations.

In the Y direction, the vertical trajectory maintained a small deviation throughout the simulation period, as shown in Figure 9. These single-axis tracking results provide a baseline reference for subsequent cross-algorithm comparisons (see Figure 10 and Figure 11), facilitating further analysis of the overall localization performance of different algorithms along the X and Y axes.

To evaluate the trajectory fitting performance and positioning accuracy of various fusion algorithms in the linear climbing task of the magnetically adhered wall-climbing robot, Figure 10 presents a comparison of trajectory errors in the X = 5 direction among three localization methods: EKF (IMU + UWB), EKF (IMU + Odom + UWB), and the proposed fusion algorithm. The results indicate that the proposed method produces relatively smaller fluctuations in the lateral trajectory and closer alignment with the ground truth, suggesting improved consistency in mitigating drift and reducing localization error under the tested conditions.

Figure 11 presents the trajectory comparison in the Y-axis direction, with local zoom-in views provided in critical regions to highlight deviations among different algorithms during vertical motion. The results suggest that EKF (IMU + Odom + UWB) shows improved continuity and tracking consistency compared to EKF (IMU + UWB), while the proposed method demonstrates relatively better overall fitting performance and smoother trajectory profiles in the tested scenario.

### 4.2. Closed Trajectory-Based Localization Simulation

To further validate the accuracy and robustness of the proposed localization algorithm under complex path conditions, a quasi-rectangular trajectory was designed as the reference path, with vertices sequentially set as (1, 1) → (5, 1) → (5, 4) → (1, 4) → (1, 1). Based on this, three sensor fusion methods were implemented and compared: (1) EKF-based fusion of an IMU and UWB; (2) EKF-based fusion of an IMU, Odometer, and UWB; (3) and the fusion algorithm proposed in this paper.

**Figure 12:**
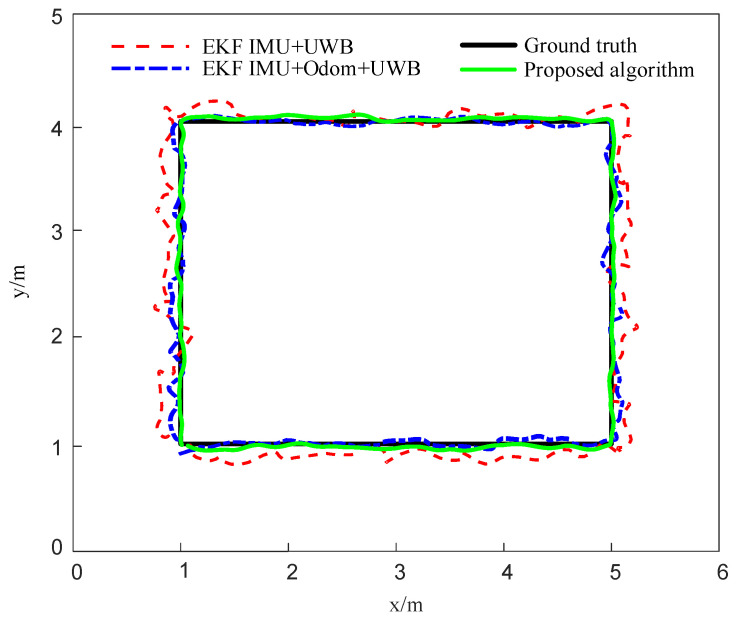
Comparison of localization trajectories along a rectangular path.

Simulation results indicate that all three estimated trajectories generally follow the reference path, though deviations are observed, particularly near corners and along straight-line segments. The EKF (IMU + UWB) method shows relatively larger deviations and fluctuations. The EKF (IMU + Odom + UWB) approach provides improved consistency by leveraging wheel odometry data. Compared with the baselines, the proposed method achieves better overall fitting, yielding a more continuous trajectory and enhanced corner-tracking stability, as illustrated in Figure 12.

To further evaluate the performance of different localization algorithms at key positions along the trajectory, four sampling points on the path were selected as comparison benchmarks. The estimated coordinate values at each sampling point for all algorithms are listed in Table 2. This table presents the single-sample estimated positions of each method, which can be used to calculate the spatial deviation of the wall-climbing robot under different localization strategies, thereby quantitatively assessing the localization accuracy of each fusion algorithm.

To mitigate the impact of random errors that may occur in a single simulation run and ensure the representativeness of the evaluation, in this study, we conducted 200 independent simulation runs. Three quantitative metrics were used to analyze localization performance: Positioning Error, Root Mean Square Error (RMSE), and Mean Error.

First, for each localization time step i, the instantaneous positioning error is defined as the Euclidean distance between the estimated position $\left(x,\;y\right)$ and the ground truth position $\left(x_{true},y_{true}\right)$. The calculation formula is as follows:

(47) $$e=\sqrt{{\left(x-x_{true}\right)}^{2}+{\left(y-y_{true}\right)}^{2}}$$

where $\left(x_{true},y_{true}\right)$ represents the ground truth coordinates of the corresponding sample point on the reference trajectory, and $\left(x,y\right)$ is the estimated position obtained by the localization algorithm at that time. e is the instantaneous positioning error at that moment.

Furthermore, to assess the overall accuracy of the localization system, the Mean Error is calculated. This metric reflects the aggregate positioning accuracy achieved by each method along the test path. A smaller mean error indicates a lower systematic deviation and higher accuracy in tracking the target trajectory. The expression is given as:

(48) $$\bar{e}=\frac{1}{n}\sum_{i=1}^{n}e_{i}$$

Meanwhile, the RMSE is introduced as a metric to quantify the fluctuation or volatility of the localization error. RMSE measures how much individual errors deviate from the overall average and is defined as:

(49) $$\sigma =\sqrt{\frac{1}{n}\sum_{i=1}^{n}{\left(e_{i}-\bar{e}\right)}^{2}}$$

where $e_{i}$ denotes the positioning error at the *i*th sampling point, and $\bar{e}$ represents the mean error across all sampling points. This metric effectively reflects the stability performance of the algorithm across different locations.

Based on the above error definitions, the positioning errors of the three fusion algorithms at key sampling points were calculated. Additionally, the Mean Error and RMSE in both the X and Y directions were statistically analyzed. These evaluations quantitatively illustrate the localization accuracy and consistency of each fusion strategy across the tested trajectories. The results are presented in Table 3 and Table 4, respectively.

According to the results in the table, the RMSE values of the EKF (IMU + UWB) method in the X and Y directions are 0.1376 m and 0.1182 m, respectively. After incorporating wheel odometry, the EKF (IMU + Odom + UWB) method reduces the errors in both directions by 38.9% and 35.5%, respectively. Furthermore, the proposed fusion algorithm further compresses the RMSE to 0.0578 m in the X direction and 0.0511 m in the Y direction under the same conditions, representing a reduction of 58.0% and 56.8% compared to the EKF (IMU + UWB) approach and 31.3% and 33.0% compared to the EKF (IMU + Odom + UWB) method. These results suggest that the proposed approach achieves improved accuracy and consistency in positioning.

**Figure 13:**
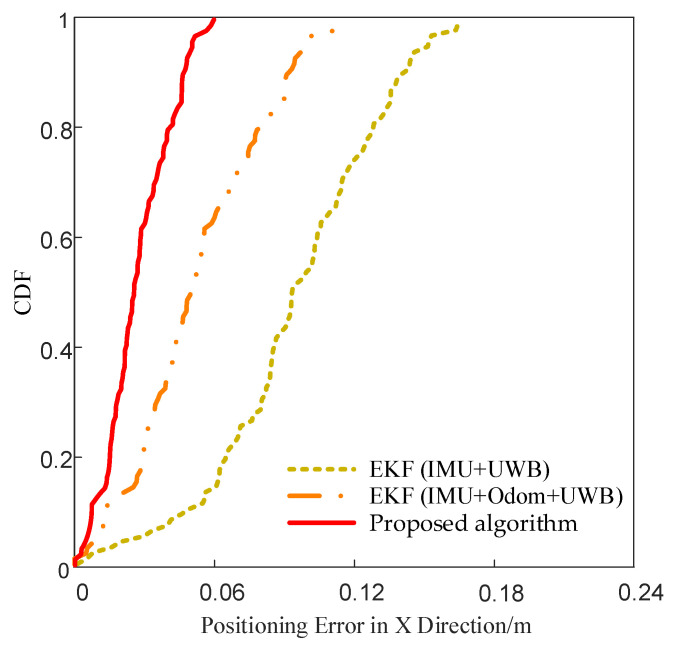
CDF comparison of X-axis positioning errors.

**Figure 14:**
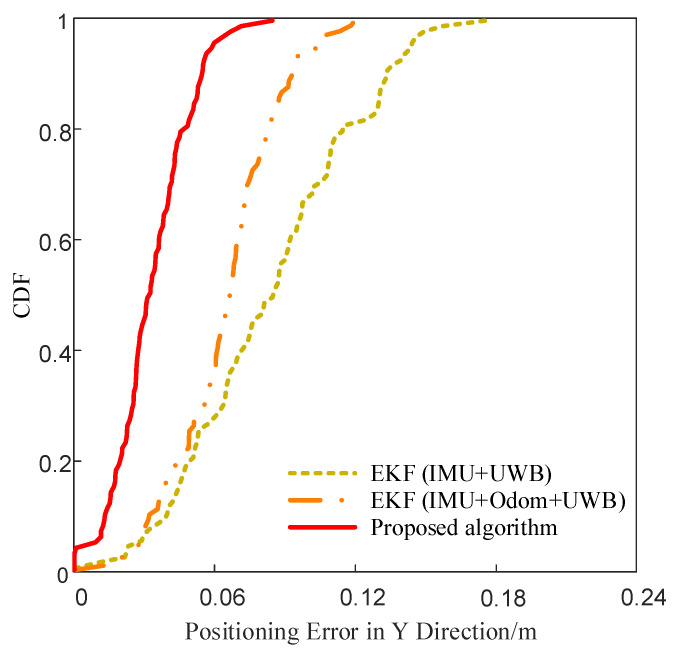
CDF comparison of Y-axis positioning errors.

To compare the error distribution characteristics of different fusion algorithms, the cumulative distribution function (CDF) curves of positioning errors in the X and Y directions were plotted for the three methods, as shown in Figure 13 and Figure 14. These curves represent the cumulative probability of error values under various thresholds. A faster convergence of the curve indicates a more concentrated error distribution and more stable localization performance.

As shown in Figure 13, the CDF curve of the proposed algorithm converges to 1 earlier than the others in the X direction, indicating higher positioning accuracy, reduced dispersion in error, and more stable performance in this axis.

### 4.3. Localization Simulation on Curved Steel Surfaces

To further evaluate the adaptability of the proposed localization algorithm on complex steel surfaces, an additional simulation was conducted on a convex spherical cap structure. This geometry mimics common industrial configurations such as the outer walls of storage tanks. The surface has a base radius of 1.5 m and a vertical height of 1.3 m.

A closed trajectory was defined at a constant height of *z* = 1 m on the surface and served as the ground truth path, represented by the black dashed line. The robot was simulated to follow this path while generating UWB, IMU, and wheel odometry data in a full three-dimensional setting; the robot’s motion in this simulation is constrained along a known reference surface. As the trajectory evolves tangentially to the surface, the proposed method can perform localization without requiring modifications to the underlying model structure.

The four UWB anchors were deployed 2 m above the four corners of the rectangular base, ensuring comprehensive spatial coverage of the curved surface. All other noise parameters and filtering settings were kept unchanged to ensure a fair comparison.

**Figure 15:**
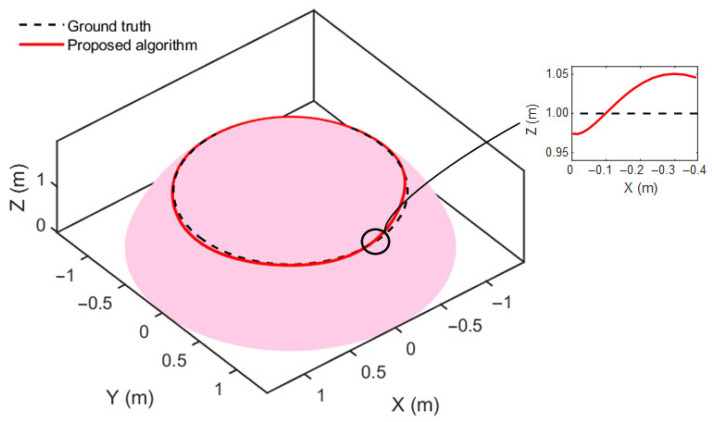
Trajectory estimation result on a curved steel surface.

The trajectory estimation result of the proposed method on the curved steel surface is shown in Figure 15, where the red curve indicates the estimated path and the black dashed line represents the ground truth. The comparison indicates that the proposed method can follow the intended trajectory under curved-surface constraints with reasonable accuracy. A magnified view shows vertical deviations along the Z-axis within approximately ±5 cm.

**Figure 16:**
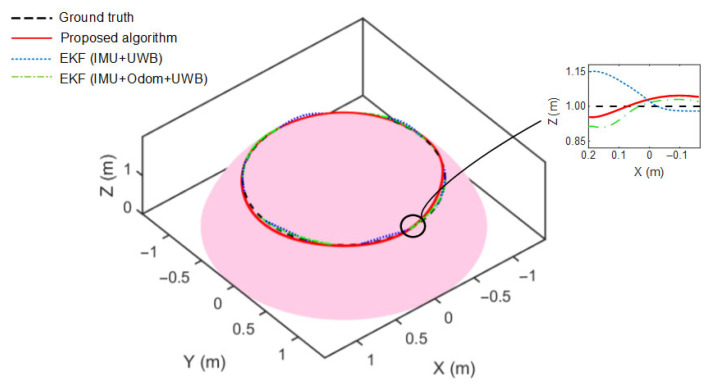
Comparison of localization methods on a curved surface.

To further evaluate the performance differences among the three localization methods under curved-surface conditions, all algorithms were applied to the same reference trajectory on a steel surface shaped as a spherical cap. As illustrated in Figure 16, the estimated trajectory generated by the proposed method shows better visual alignment with the ground truth compared to the other two EKF-based strategies. The inset view highlights a segment with observable vertical deviations. Among the three methods, the one using only an IMU and UWB exhibits relatively larger fluctuations in Z-axis estimation, while incorporating odometry results in moderate improvement. In comparison, the proposed method yields relatively smaller vertical deviations, suggesting improved consistency in vertical position estimation under curved-surface constraints.

## 5. Conclusions

To address the challenges of positioning error accumulation and insufficient robustness in magnetically adhered wall-climbing robots operating on large steel structures, this paper presents a multi-sensor fusion localization method integrating an IMU, Odom, and UWB. The proposed framework incorporates a complementary filter for high-frequency motion prediction and introduces a residual-weighted optimization mechanism for UWB ranging data, enhancing the robustness of state estimation under nonlinear and imperfect conditions via an EKF.

Comprehensive simulations were conducted under multiple conditions, including linear motion, quasi-rectangular closed loops, and curved-surface trajectories. The results consistently demonstrate that the proposed method outperforms traditional EKF-based fusion schemes (IMU + UWB and IMU + Odom + UWB) in both accuracy and stability. Specifically, average positioning errors in the X and Y directions are reduced to approximately 4.1 cm and 4.6 cm, respectively, while CDF analysis reveals improved error concentration and faster convergence. Furthermore, in curved-surface scenarios designed to emulate realistic 3D operating conditions, the proposed algorithm achieves improved trajectory consistency and reduced drift along the Z-axis.

In future work, we will aim to enhance the method’s localization performance in more challenging environments by addressing factors such as dynamic external disturbances and environmental complexity and expanding the localization capabilities to handle more complex scenarios. Furthermore, we plan to integrate vision-based perception and data-driven fusion strategies (e.g., deep learning-assisted filtering) to improve environmental awareness and positioning robustness and support long-term autonomous operation in complex steel structure inspection tasks.

## Figures and Tables

**Table 1:** Main configuration parameters for the simulation.

Parameter	Value
ch1, ch2, ch3, ch4	(0, 0), (10, 0), (10, 10), (0, 10) m
v	0.1 m/s
ω	0 rad/s
θ	0 rad
T	0.01 s
α	0.7
β	0.3
γ	0.6
b	$0.02\;m/s^{2}$
n	0.01 rad/s
r	0.06 m

Note: ch1-4 are the coordinates of the base stations, *v* is the linear velocity, *ω* is the angular velocity, *θ* is the heading angle, *T* is the time step, *α*, *β*, and *γ* are the complementary filter weighting coefficients, *b* is the sensor bias, *n* is the sensor white noise, and *r* is the radius of the magnetic adhesion wheel.

**Table 2:** Trajectory sampling coordinates at key positions.

Number	Sample Point	EKF IMU + UWB	EKF IMU + Odom + UWB	Proposed Algorithm
1	(1.000, 1.000)	(1.124, 1.093)	(1.080, 1.066)	(1.046, 0.973)
2	(5.000, 1.000)	(4.891, 1.087)	(5.083, 0.914)	(4.962, 1.068)
3	(5.000, 4.000)	(5.126, 4.118)	(5.070, 4.082)	(5.049, 4.051)
4	(1.000, 4.000)	(0.870, 3.883)	(0.926, 3.955)	(1.052, 3.952)

**Table 3:** Mean Positioning Error.

Positioning Method	Mean Error in the X Direction (m)	Mean Error in the Y Direction (m)
EKF (IMU + UWB)	0.1304	0.0962
EKF (IMU + Odom + UWB)	0.0741	0.0688
Proposed algorithm	0.0462	0.0503

**Table 4:** Root Mean Square Errors.

Positioning Method	Mean Error in the X Direction (m)	Mean Error in the Y Direction (m)
EKF (IMU + UWB)	0.1376	0.1182
EKF (IMU + Odom + UWB)	0.0841	0.0763
Proposed algorithm	0.0578	0.0511

## Data Availability

The simulated trajectories supporting the analysis are available from the authors upon reasonable request and subject to compliance with relevant regulations.
